# Moisture Content Estimation of Porous Building Stones using Hyperspectral Imaging

**DOI:** 10.1038/s41597-025-06416-4

**Published:** 2025-12-09

**Authors:** Danish Ali Chaghdo, Bikram Koirala, Laura Cristina, Tim De Kock, Laurent Fontaine, Roald Hayen, Paul Scheunders

**Affiliations:** 1 Monuments and Monumental Decoration Lab, Royal Institute of Cultural Heritage (KIK-IRPA), JubelPark 1, Brussels, 1000 Belgium; 2 Visionlab, Department of Physics, University of Antwerp, Universiteitsplein 1, Antwerp, 2610 Belgium; 3 Department of Physics ”G. Occhialini”, University of Milano-Bicocca, Piazza della Scienza, 3, Milan, 20126 Italy; 4 Antwerp Cultural Heritage Sciences, University of Antwerp, Mutsaardstraat 31, Antwerp, 2000 Belgium

**Keywords:** Computer science, Scientific data

## Abstract

Moisture poses a major threat to built heritage through processes such as frost damage, salt crystallization and biological growth, which accelerate stone deterioration. Stone moisture content can be determined non-destructively by spectral reflectance in the shortwave infrared (SWIR) wavelength range. To validate this, large cubic and small cylindrical samples from six stone types, Brick, Euville, Massangis, Neubrunn, Obernkirchen, and Savonnières, with different controlled moisture levels were prepared, and a comprehensive SWIR hyperspectral image dataset was created. The acquired hyperspectral images were processed using the Normalized Relative Arc Lengths (NRAL) method to generate moisture maps that illustrate the spatial distribution of water within each sample. Because moisture distribution is influenced by pore characteristics and grain arrangement, the moisture maps were validated through petrographic examination. For quantitative validation, the mean moisture content of each sample was compared with the corresponding gravimetric moisture content. The results demonstrated strong agreement between the estimated and gravimetric moisture values, with root mean square errors ranging from 1 and 2 g/g  × 100.

## Background & Summary

Built heritage (BH) is considered important because it serves as an expression of the cultural identity of cities and communities, providing tangible evidence of the past^1^. Due to the immense value of BH, its preservation for future generations is a collective responsibility^2^. This effort typically follows a structured approach that involves analysis, diagnosis, and continuous monitoring in order to determine appropriate intervention strategies. Among the various natural degradation processes affecting building materials, water stands out as one of the major drivers for deterioration^3,4^. Several undesirable phenomena are related to water, such as frost damage^5^, salt crystallization^6^, and biological growth^7^, making water a concrete threat to architectural heritage^8^. Because the preservation of BH is a major concern^2^, detecting the presence of moisture and monitoring it over time is of considerable importance for its maintenance^4^.

Direct moisture measurement methods, of which gravimetric and calcium carbide methods are the most popular, are generally considered the most reliable for quantitative measurement of moisture content (MC) in building elements through appropriate sampling^9^. However, these techniques require sample collection and hole drilling. Sampling is usually carried out using a powder form drill or as a core sample of approximately 2 cm in diameter and as deep as necessary. This is an invasive procedure that may pose a risk of damage to historical materials. In addition, due to the heterogeneous distribution of water, sampling should be repeated several times at different positions, which further increases the destructive character^4^. The MC is then assessed by determining the mass of water in the samples (gravimetric technique)^10^ or measuring the pressure caused by a chemical reaction and correlating the pressure with the MC (calcium carbide technique)^4,9^. Although these methods are accurate and the results are reliable^9^, especially gravimetric analysis^11^, they are not recommended for application to BH materials due to their destructive approach^12^. Furthermore, given the destructive nature of the analysis, sampling points are often chosen to minimize the damage caused by the technique, with the risk of unrepresentative results^13^.

Over the years, non-destructive methods have been proposed to overcome physical sampling^14^. Within non-destructive techniques, two different categories can be distinguished: point-based techniques and imaging techniques. Point-based methods, such as ultrasonic testing^15^, moisture meters^16^, and microwave systems^17^, provide local information by analyzing specific points in the material^12^. While these methods are effective for localized analysis, point techniques often fail to capture broader patterns of damage or moisture distribution^12^. Since the main objective is to determine MC, imaging techniques could provide more representative results and indicate the best spots for local analysis^18^. The study of BH by non-invasive imaging analysis provides a preliminary screening of the object under investigation and a comprehensive evaluation of its surface. A correct analytical approach will then include, if necessary, the use of non-invasive analytical point-based techniques^19^. Among imaging techniques, Infrared Thermography (IRT) has been widely used for the detection of moisture^20^, because it is a non-destructive technique and allows *in situ* measurements^21^. Because changes in MC correlate with changes in surface temperature, IRT can be used to map moisture distribution and identify areas of atypical moisture content. However, the quantitative analysis of surface temperature differences in relation to the MC of walls remains challenging due to the complex and variable relationship between evaporative flow and surface temperature^22^. The Earth Resistivity Tomography (ERT) method provides valuable insights into the distribution of moisture within historic stone structures, allowing for both 2D and 3D imaging. However, compared to high-resolution techniques, ERT has lower spatial resolution, which may limit the accuracy and detail of moisture mapping in these materials^23^. Advanced laboratory techniques have been used for more detailed analysis of moisture content and moisture transport. With portable nuclear magnetic resonance (NMR) sensors, the distribution of moisture in historic walls can be mapped^24,25^. This method is not applicable in the presence of magnetic fields, and organic materials may distort the results^4^. Neutron scattering-based techniques in two (radiography^26,27^) and three dimensions (tomography^28^) have high accuracy in water content estimation but lower spatial resolution compared to other attenuation techniques^4^.

Over the last years, there has been a growing interest in hyperspectral imaging (HSI) techniques^18^, with several applications in the domain of BH^29^. HSI was introduced into the domain of BH in the 1990s and has continued to improve since then, with an increase in the quality of spectroscopic information in acquired imaging data^19^. This technique consists of acquiring images in a sufficient number of contiguous spectral bands. As a result, each pixel in the image has an associated reflectance spectrum. The collected dataset typically includes hundreds of images acquired in narrow spectral bands (bandwidth: 2-10 nm) and recorded over an extended spectral range^19,30^, spanning from the visible and near-infrared (VNIR, 400-1000 nm) to the shortwave-infrared (SWIR, 1001-2500 nm) range. HSI offers significant potential for the quantitative evaluation of MC in building materials because water exhibits strong absorption, especially in the SWIR range (e.g., absorption peaks around 1400 and 1900 nm). In these wavelength ranges, the optical reflectance properties of water-bearing materials are mainly influenced by water. Despite the promising potential of HSI for MC monitoring^31^, the application of this technique in the context of BH remains significantly limited^32,33^.

In contrast, over the past two decades, researchers in the remote sensing community have exploited the potential of HSI to estimate soil MC, leveraging its ability to provide large-scale, non-contact, and timely assessments^34^. The methodologies developed to quantitatively estimate soil MC can be grouped into empirical methods^35^ and methods based on physical modeling^36^. Empirical methods include spectral indices, statistical relationships, exponential functions, wavelet analysis, and multivariate analysis^37^. Physical methods aim to describe the physical interaction between light and soils, with the advantage that it allows simulation of the reflectance spectrum of moist soils^37^.

It is a well-known fact that variations in illumination and viewing angles significantly impact the measured reflectance spectra. Additionally, the measured reflectance of moist soil highly depends on the soil grain size and grain size distribution. Existing empirical and physical methods cannot efficiently address these challenges.

Recently, an efficient quantitative method for soil moisture estimation has been proposed^37^. The proposed method generates a proxy for soil MC by expressing the reflectance of a moist sample relative to that of both a dry and a saturated sample. This proxy is then normalized by the MC of the saturated soil sample to obtain an estimate of the MC of the moist sample. The approach ensures invariance to variations in illumination, viewing angles, and soil type. Since this method’s effectiveness has already been demonstrated, it has been adopted in this study to produce moisture data for stone samples obtained from BH. The remainder of this manuscript is organized as follows. Section 2 describes the materials used, the sample preparation, and the acquisition setup. Data records are described in Section 3. Section 4 presents a technical validation of the moisture data, which includes a comparison of the moisture data with polarized microscopic images obtained by petrographic examination and scatterplots comparing the mean moisture data of each sample with the gravimetric moisture content.

## Methods

### Material Description

In this study, we selected representative porous materials, limestone, sandstone, and brick, commonly used in construction, while excluding dense or clay rich rocks. The selection aimed to cover different mineralogies (siliciclastic rocks, carbonate rocks, and bricks with fired/calcined clay) and a range of pore structures (unimodal and multimodal). Six samples, Euville, Neubrunn, Savonnières, Massangis, Obernkirchen, and Brick, were chosen to represent the heterogeneity of natural stones in accordance with EN 1936 and EN 1926 standards. Euville is a crinoidal limestone of Upper Jurassic age from Commercy, France (48.750° N, 5.625° E); Massangis is a bioclastic oolitic limestone of Middle Jurassic age from Massangis, France (47.624° N, 3.973° E); Neubrunn is a sandstone of Upper Triassic age from Neubrunn, Germany (49.731° N, 9.672° E); Obernkirchen is a sandstone of Early Cretaceous age from Obernkirchen, Germany (52.264° N, 9.130° E); Savonnières is an oolitic limestone of Upper Jurassic age from Savonnières-en-Perthois, France (48.606° N, 5.132° E). Additionally, a red brick excavated from the Kipdorp archaeological site in Antwerp, Belgium (51.2199° N, 4.4159° E), was included in the study.

To account for material heterogeneity, six replicate specimens were prepared for each stone type, such that the experimental data set consisted of 36 specimens in total: 30 natural stone samples (5 stone types  × 6 replicates) and 6 brick samples. From the natural stones, cubic samples of size (6 × 6 × 6 cm^3^) were prepared, while the archaeological brick samples exhibited more irregular dimensions averaging 3.5 × 5.5 × 6.5 cm^3^ but also having one fresh cut, flattened surface.

To assess the local spatial homogeneity or heterogeneity of the material, small cylindrical samples (nominal diameter 10 mm, height 20 mm) were extracted from each of the 36 larger samples using wet core drilling, followed by flattening the top and bottom surfaces with a stone cutter. These dimensions of smaller cylindrical samples are chosen with the understanding that, in future work, these samples will be utilized to assess the pore size distribution of stone samples through Mercury Intrusion Porosimetry (MIP).

All samples were labeled as big or small B (red brick), E (Euville), M (Massangis), N (Neubrunn), OB (Obernkirchen) and S (Savonnières), along with a number (1 to 6) to denote the specific replicate.

### Sample Preparation

Firstly, some preliminary tests have been carried out to evaluate the drying process of the different types of stones, and the time required for complete drying. The samples were oven-dried at 60°C for 24 hours. It is important to note that drying at 60 °C for 24 hours was chosen because it is a well-established in-house procedure at KIK-IRPA, a federal scientific institute in Belgium, where it has been routinely applied for several years in stone and mortar characterization studies. The dry condition was reached when the change in weight between repeated measurements was considered negligible. The weights were measured with an analytical balance (readability 0,01g)

**Fig. 1:**
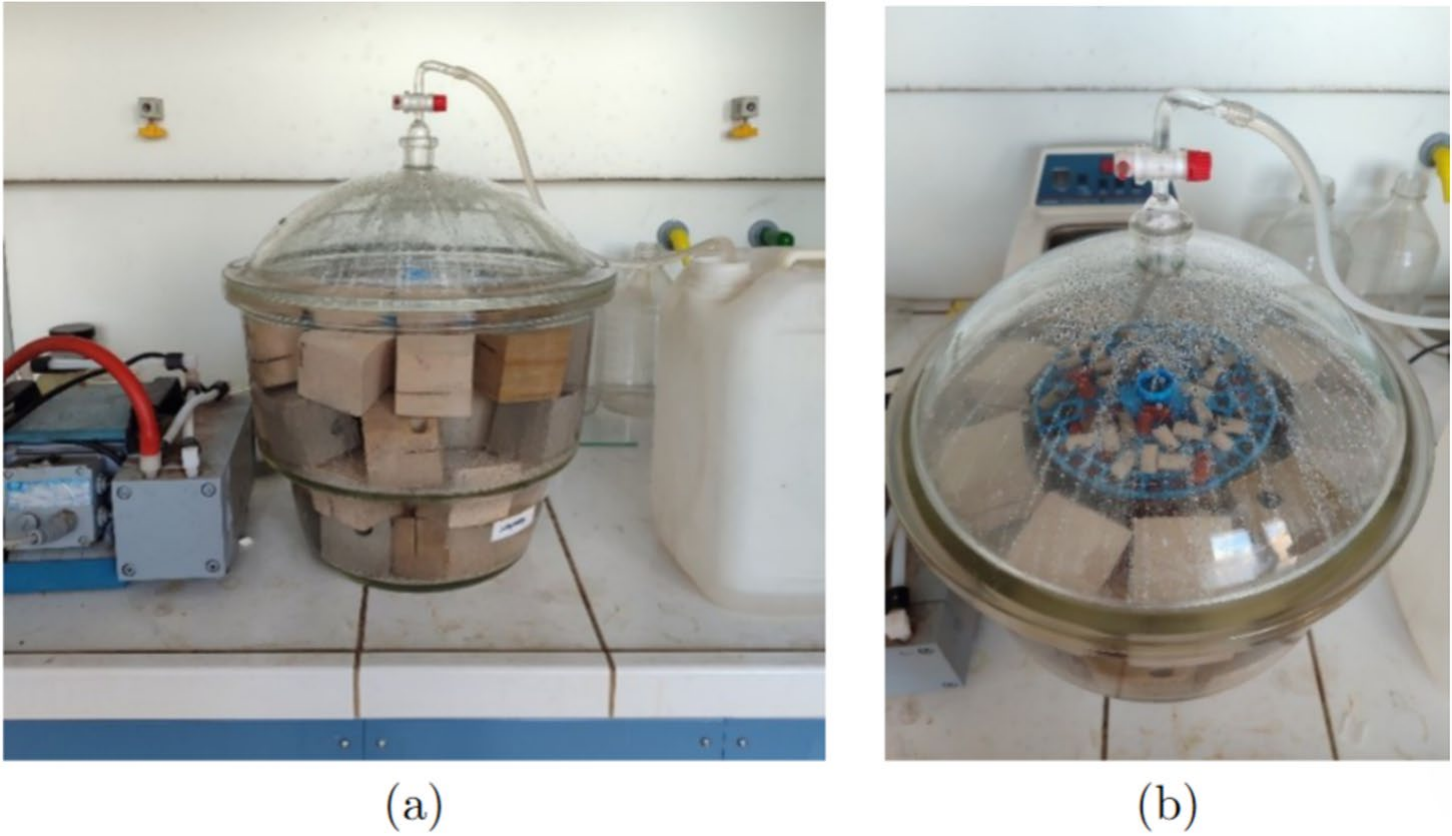
(**a**) Stone samples are placed inside a desiccator, and water is gradually introduced through a pipe inside the desiccator after removing the air with a vacuum pump. (**b**) Disposition of the stone samples inside the desiccator.

Samples were vacuum saturated under the following procedure:

- The stone samples are placed inside a exsiccator connected to a vacuum pump.
- After 2 hours of vacuum drying, water is gradually introduced into the exsiccator through a dedicated inlet.
- After full immersion, the samples are left in water for 4 days, a period considered sufficient for the water to fully penetrate the pores of the stones (see Fig. 1). The samples are assumed to be fully saturated (saturation 100%).

Different degrees of MC, relative to saturation were considered to obtain a representative picture of the phenomenon. The selected degrees of saturation were: 25%, 50%, 75%, 90% and 100%. The 90% saturation level was considered to eventually evaluate a non-linear behavior of the measurements close to the saturation level. Moreover, any liquid water present on the surface of the fully saturated samples was carefully removed with a damp cloth to minimize its influence on the measured spectral reflectance. To calculate the masses at *x* = 25%, 50%, 75% and 90% degree of saturation, the following equation was used:

1 $${m}_{x \% }={m}_{d}+\frac{x}{100}({m}_{100 \% }-{m}_{d})$$

where *m*_*x*%_ is the mass of the sample at x% saturation level, *m*_*d*_ is the mass of the dry sample and *m*_100%_ is the mass of the saturated sample. The MC of a sample will be expressed as:

2 $$\,{\rm{MC}}\,(x \% )=\frac{({m}_{x \% }-{m}_{d \% })}{{m}_{d \% }}\times 100$$

(in g/g  × 100).

The different saturation levels are obtained by drying the samples in the oven (60^∘^C) until they reach the correct calculated mass. The mass of the sample was continuously monitored to achieve the desired saturation level. The time interval between two different mass measurements was approximately 5 min. For small samples, mass differences between percentage levels were small (between 0.06 and 0.21 g). Therefore, in a few cases, it was necessary to add some water with a pipette when the drying process in the oven resulted in a weight lower than the calculated one.

Hyperspectral imaging only captures information about the sample’s top surface. For the bulk water content to align with the averaged hyperspectral estimation over the entire surface, the water must be evenly distributed throughout the sample. Sample homogeneity is essential so that the obtained HSI results can be considered as information from the bulk. Therefore, samples were wrapped in plastic films after obtaining the target weight and kept for minimum 48 hours to obtain equilibrium water distributions while avoiding water loss through evaporation. Two sheets of plastic were used to wrap the sample. This process was carefully repeated for all samples. After hyperspectral imaging, all samples were reweighed with an analytical balance to assess any mass loss. This operation was important to check that the same level of saturation was maintained.

### Hyperspectral Image Acquisition Setup

In this work, we used a Snapscan SWIR hyperspectral camera manufactured by Imec to acquire hyperspectral images of the prepared samples. The Snapscan SWIR camera operates over a spectral range of 1120.5-1675.1 nm, capturing 100 spectral bands with a spectral resolution varying from 0.32 nm to 15.37 nm and a mean interval of approximately 5.6 nm.

All samples were positioned consistently and scanned from the same side to ensure uniformity in data collection. Four halogen lamps, placed at a 45-degree angle to the hyperspectral camera, provided uniform illumination to reduce shadows and increase brightness. Unlike traditional push broom systems, the Snapscan camera uses an internal sensor motion mechanism to capture a static, full image frame of the sample, improving spatial resolution and simplifying the setup. The original frame size of the raw images was 400 × 400 pixels for the larger samples. Since these images included both the sample and the background, the hyperspectral cubes were cropped to remove background pixels, resulting in final image cube sizes ranging from 286 × 210 × 100 to 391 × 384 × 100.

**Fig. 2:**
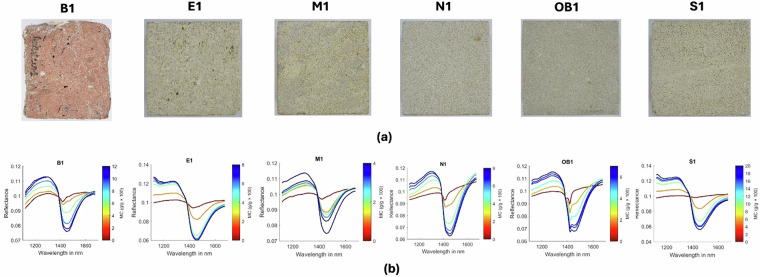
(**a**) RGB images of the stone samples: (**b**) measured reflectance spectra for different MC levels and corresponding ground truth MC of stone samples.

Figure 2 presents an RGB image of one representative sample from each stone type, together with the corresponding mean reflectance spectra and ground-truth MC for the different moisture levels. It can be observed that the reflectance spectra of moist samples are primarily influenced by water, which plays a critical role in enabling accurate estimation of the MC of porous materials. However, significant differences in the measured spectra are observed among different stone samples due to variations in pore size and distribution. Therefore, to accurately predict moisture content from the measured spectra, a unique representation of the moist sample that is invariant to the material’s pore structure must be determined.

### Generation of moisture data

To determine a unique representation of moist samples that is invariant to variations in pore size and distribution, and to predict the moisture content, in^37^, a method called Normalized Relative Arc Lengths (NRAL) was proposed. The method is expected to be applicable to all porous materials (e.g., soil, clay, stone, wood, etc.). In this work, we applied this method to generate moisture data from the acquired spectral reflectance data of the stone samples. NRAL assumes that the data manifold generated by multiple moist samples with varying MC values forms a curve connecting the spectral reflectances of air-dried and saturated samples (the endmembers). Estimating MC thus becomes a matter of determining a moist sample’s relative position along this curve.

To accurately determine the relative position of a sample, the length of the curve must be appropriately approximated. Since the curve between endmembers can be represented as a piecewise linear curve, its total length can be estimated by summing the Euclidean distances between consecutive intermediate points. Increasing the number of intermediate points improves the accuracy of this approximation.

However, in many practical cases, only a single spectrum of the moist sample is available for estimating its MC. In such scenarios, the approximated curve length becomes unreliable. To overcome this limitation, each spectrum can be projected onto the unit hypersphere, where the arc length between any two spectra (e.g., the endmembers) corresponds to the angle between them. This angle can be computed as the arc cosine of their dot product. The projection onto the unit hypersphere is achieved by normalizing each spectrum by its magnitude: (${\bf{y}}\to \frac{{\bf{y}}}{\left\Vert {\bf{y}}\right\Vert }$). An additional advantage of this representation is that the unit hypersphere space is invariant to variations in illumination and acquisition geometry.

**Fig. 3:**
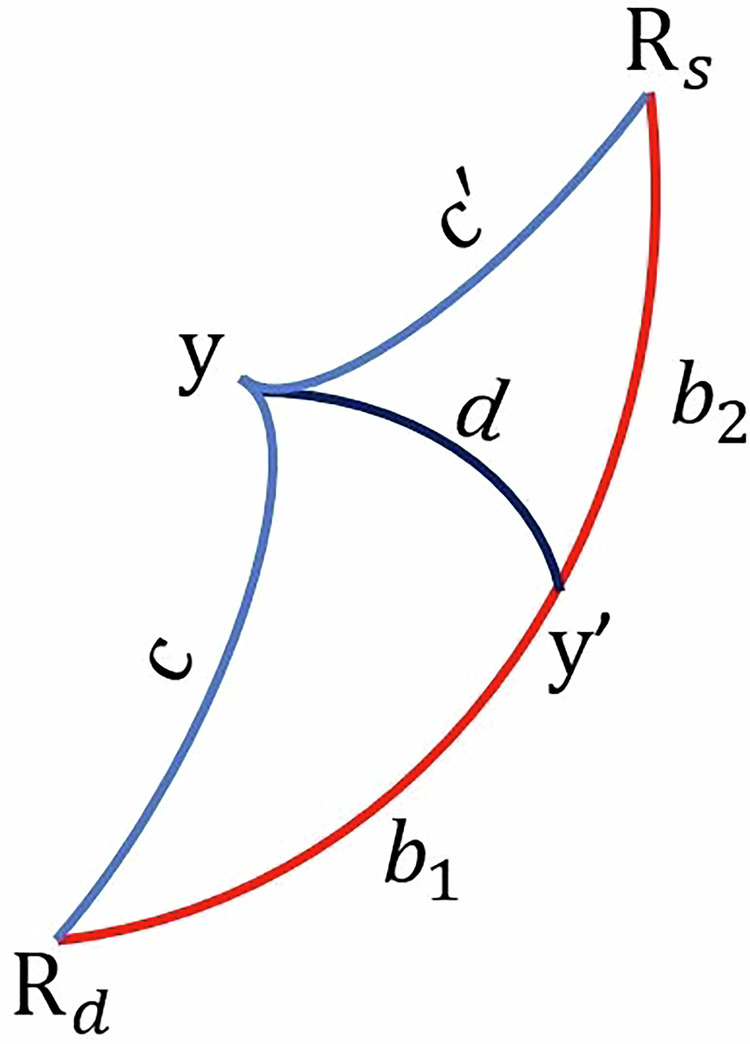
Red curve: the arc connecting the dry endmember **R**_*d*_ and the saturated endmember **R**_*s*_; Blue curves: the arcs connecting the moist soil (**y**) with the endmembers. *c* and $c{\prime}$ denote the arc lengths between **y** and the endmembers, respectively. ${{\bf{y}}}^{{\prime} }$ denotes the projection of **y** on the arc connecting the endmembers, and *b*_1_ and *b*_2_ denote the true arc lengths between ${{\bf{y}}}^{{\prime} }$ and the endmembers (adapted from^37^).

Nevertheless, even after projecting the spectra onto the unit hypersphere, the spectral reflectance of a moist sample does not necessarily lie precisely along the arc connecting the two endmembers (see Fig. 3). To address this limitation, NRAL employs the spherical law of cosines (see^37^ for a detailed explanation):

3 $$\cos {b}_{1}=\frac{\sin ({b}_{1}+{b}_{2})}{\sqrt{{\left(\frac{\cos {c}^{{\prime} }}{\cos c}-\cos ({b}_{1}+{b}_{2})\right)}^{2}+{\sin }^{2}({b}_{1}+{b}_{2})}}$$

where ${b}_{1}+{b}_{2}=\arccos ({R}_{d}^{T}{R}_{s})$. The relative position of the moist sample along the curve is calculated as the ratio between *b*_1_ and the sum (*b*_1_ + *b*_2_), expressed as $\widehat{a}={b}_{1}/({b}_{1}+{b}_{2})$

In the final step, the relative position of the moist sample can be calibrated against the MC of the saturated sample (*m*_100%_) to establish the following relationship:

4 $$\,{\rm{MC}}\,(x \% )=\widehat{a}\times \,{\rm{MC}}\,(100 \% )$$

#### Moisture Maps

**Fig. 4:**
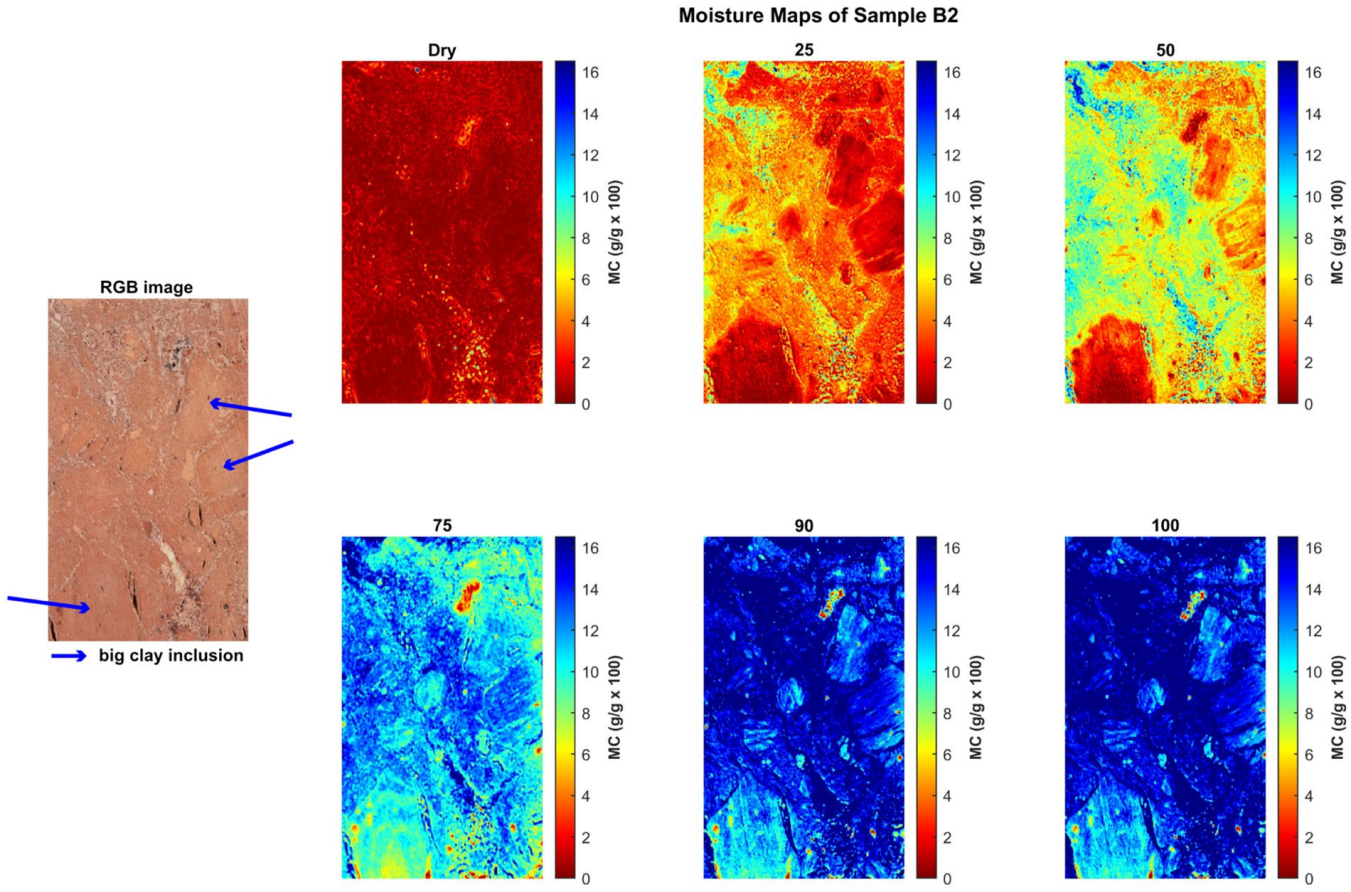
Moisture maps of brick sample B2 at varying moisture levels, with corresponding RGB image of the area of interest.The arrows show big clay inclusions comparable to those described in subsection 4.2.

**Fig. 5:**
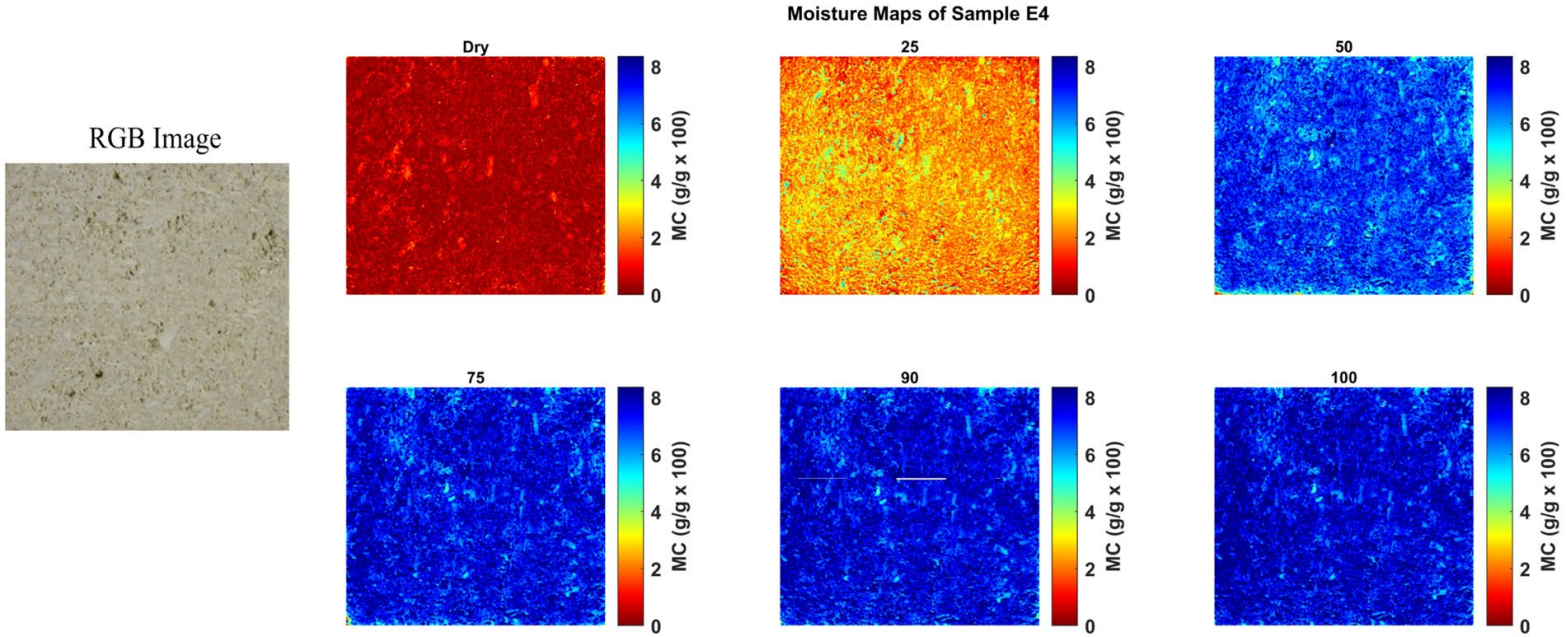
Moisture maps of Euville sample E4 at varying moisture levels, with corresponding RGB image of the area of interest.

**Fig. 6:**
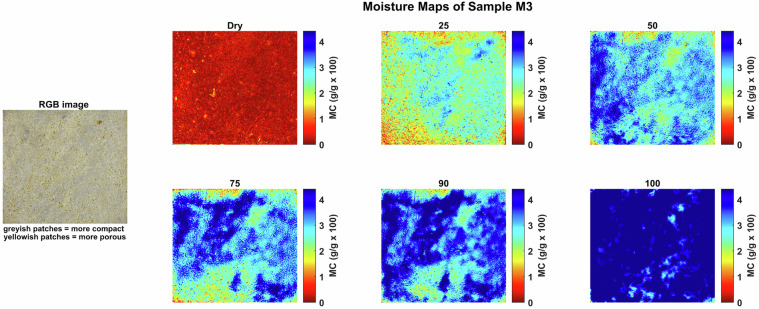
Moisture maps of Massangis sample M3 at varying moisture levels, with corresponding RGB image of the area of interest.

**Fig. 7:**
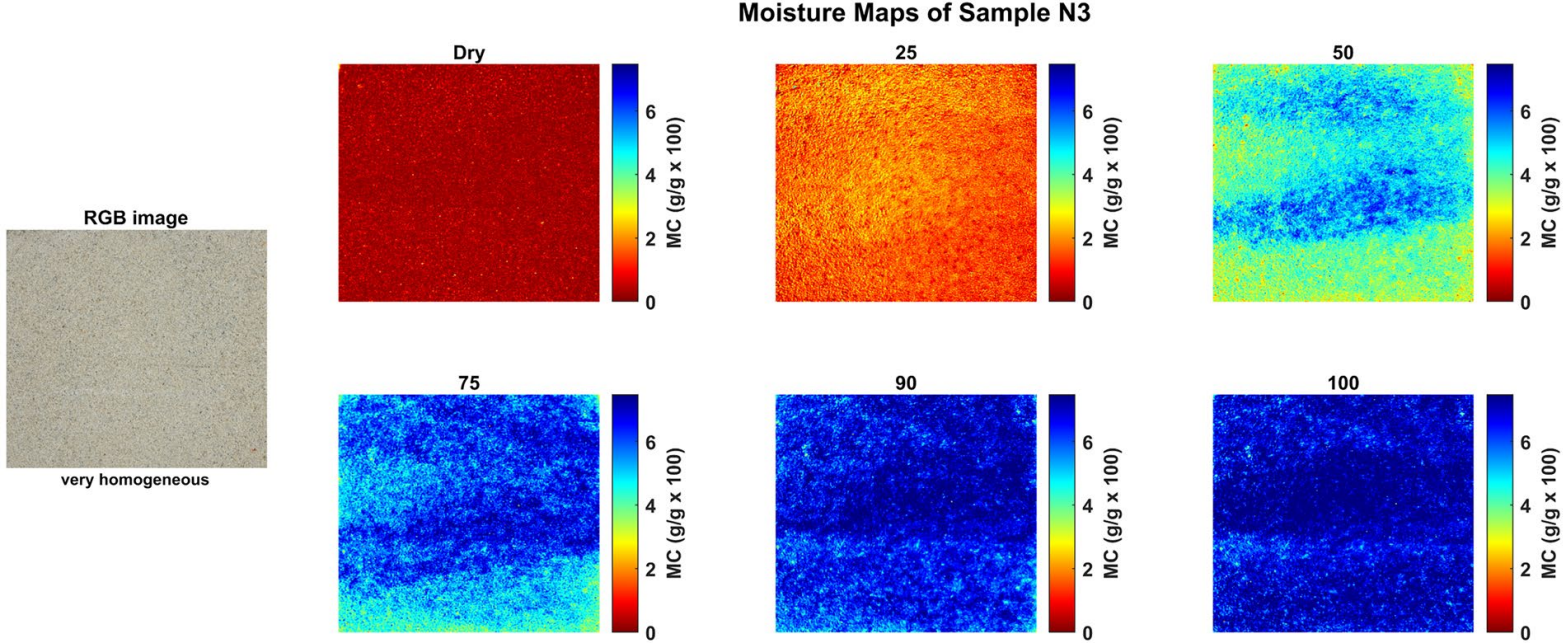
Moisture maps of Neubrunn sample N3 at varying moisture levels, with corresponding RGB image of the area of interest.

**Fig. 8:**
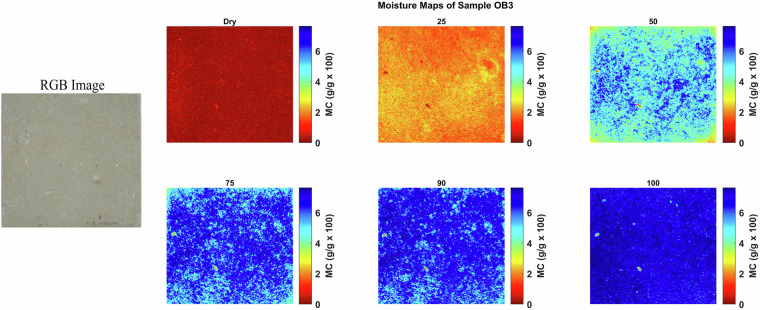
Moisture maps of Obernkirchen sample OB3 at varying moisture levels, with corresponding RGB image of the area of interest.

**Fig. 9:**
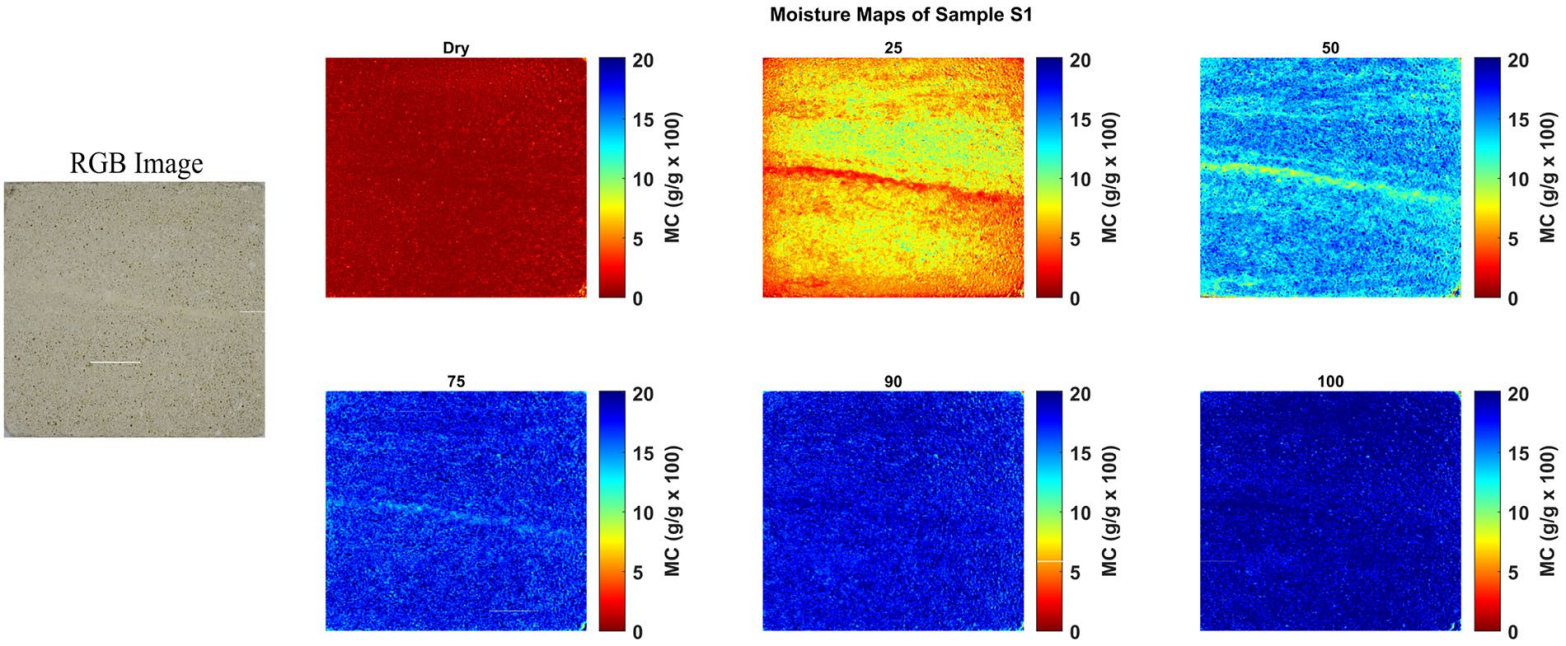
Moisture maps of Savonnières sample S1 at varying moisture levels, with corresponding RGB image of the area of interest.

Moisture maps were generated by applying the NRAL method to each hyperspectral pixel in order to visualize the spatial distribution of moisture within the stone samples. These maps help to identify zones of varying moisture content and provide insights into the absorption and retention behavior of the materials. These maps show that at any given bulk moisture content, the distribution of the moisture can be quite heterogeneous on a smaller scale, reflecting the rock texture. Figures 4, 5, 6, 7, 8 and 9 present the moisture maps for the big samples B2, E4, M3, N3, OB3 and S1 respectively, at different stages of moisture content.

In Brick, the moisture maps (sample B2), as shown in Fig. 4, show moisture first filling the large, well-connected shrinkage cracks (porosity zone 1) at 25% MC, followed by progressive infiltration at 50% and 75% MC into the clay matrix (porosity zone 2), with the clay-rich inclusions (porosity zone 3) absorbing the lowest relative amount of moisture at each step. Despite near-full saturation at 90% and 100% MC, some inner parts of clay-rich inclusions remained relatively dry, indicating limited effective porosity.

The moisture maps of Euville limestone, as shown in Fig. 5, indicate that the stone is virtually dry at the initial state. At 25% MC, water is mainly located in the most accessible pores, corresponding to the intergranular pore network forming a continuous system of interconnected voids (highlighted in yellow and green on the maps). From 50 to 100% MC, the water is evenly distributed throughout the stone.

The moisture maps of Massangis limestone (sample M3), as shown in Fig. 6 show that moisture is present in the stone at relatively low MC (25%), but preferentially in certain zones. Although these zones of higher moisture content are gradually expanding at 50, 75 and 90% MC, there remain small zones with a lower moisture content at full saturation level (100% MC). In the case of Massangis, the distribution of moisture can be explained by the microstructure of the stone. Indeed, water preferentially occupies yellowish patches, the greyish patches being less absorbent, as can been seen on the close-up of the stone surface (RGB image of M3 on the left in Fig. 6.

For Neubrunn sandstone (sample N3), as shown in Fig. 7, the moisture distribution initially appeared homogeneous at low saturation level (25% MC), but began to reflect existing laminae in the stone at higher MCs. The presence of these laminae is typical of sedimentary sandstones; however, they were not clearly visible in the physical samples. We can suppose that water accumulated preferentially in laminae with slightly larger and/or slightly better-connected pores at 50% and 75% MC. At near-full saturation level (90%–100% MC), the laminae with lower initial absorption contained even slightly more water than the others, possibly due to a higher content in the finest pores enhancing capillary suction power.

The moisture maps of Obernkirchen sandstone, as shown in Fig. 8, indicate that the stone is virtually dry at the initial state. At 25% MC, water is evenly distributed, primarily within the intergranular pores. At 50% MC, the water remains evenly distributed throughout the stone. Similarly, at 75 and 90% MC, the distribution shows little to no difference compared with lower saturation levels. At full saturation (100% MC), the stone is uniformly moist, indicating complete saturation of the accessible pore network.

The moisture maps of Savonnières limestone, as shown in Fig. 9, indicate that the stone is virtually dry at the initial state. At 25 and 50% MC, water is unevenly distributed, reflecting subtle differences in pore size and cementation between successive sets of cross-laminae; less cemented laminae appear to absorb moisture more readily than the more compact ones. At 75 and 90% MC, the water distribution becomes more uniform, with only minor differences between these two saturation levels. At full saturation (100% MC), moisture is evenly distributed throughout the stone, with some laminae that were initially less absorbent now appearing as the most moisture-rich zones, likely due to capillary condensation effects.

## Data Records

A dataset comprising hyperspectral images and corresponding moisture maps from six porous building materials is available on Zenodo^38^.

## Technical Validation

In this section, we evaluate the moisture dataset using two complementary approaches. The first approach quantitatively compares the mean moisture content of each sample, as estimated by the NRAL method, with the gravimetric moisture content of the bulk sample using scatterplots. The second approach qualitatively compares the moisture distribution on the top surface of each sample with polarized microscopic images obtained through petrographic analysis of thin sections, which reveal the pore characteristics and grain arrangements of the stone samples. The following subsections describe these two validation methods in detail.

### Scatterplot Analysis

**Fig. 10:**
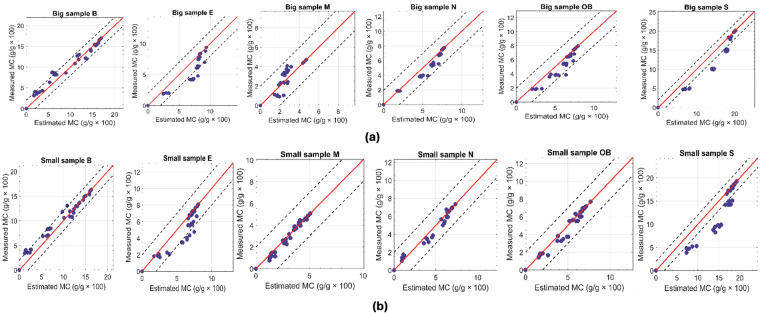
Measured vs Estimated MC of stones: (**a**) Top row: results from the big cubic samples; (**b**) Bottom row; results from the small cylindrical samples.

Figure 10 presents scatterplots comparing the mean moisture content of each sample with the gravimetric moisture content. The results include data from both large cubic and small cylindrical samples across all six stone types and moisture levels. The error in the moisture content, expressed as the root mean squared error (RMSE), ranges from 1 to 2 g/g  × 100. As expected, the smaller samples exhibit slightly higher precision in moisture content values than the larger ones, primarily due to their lower spatial heterogeneity. For highly heterogeneous samples, such as sample M, the results are notably improved for the smaller samples. Samples E and S remain heterogeneous even at smaller scales, with both wet and dry regions coexisting within the samples, which explains the relatively higher RMSE.

### Petrographic analysis

Since the distribution of moisture within a sample depends on its pore characteristics and grain arrangement, a detailed petrographic examination was conducted. The polarized microscopic images obtained through this analysis were used to validate the moisture data derived from the hyperspectral images.

**Fig. 11:**
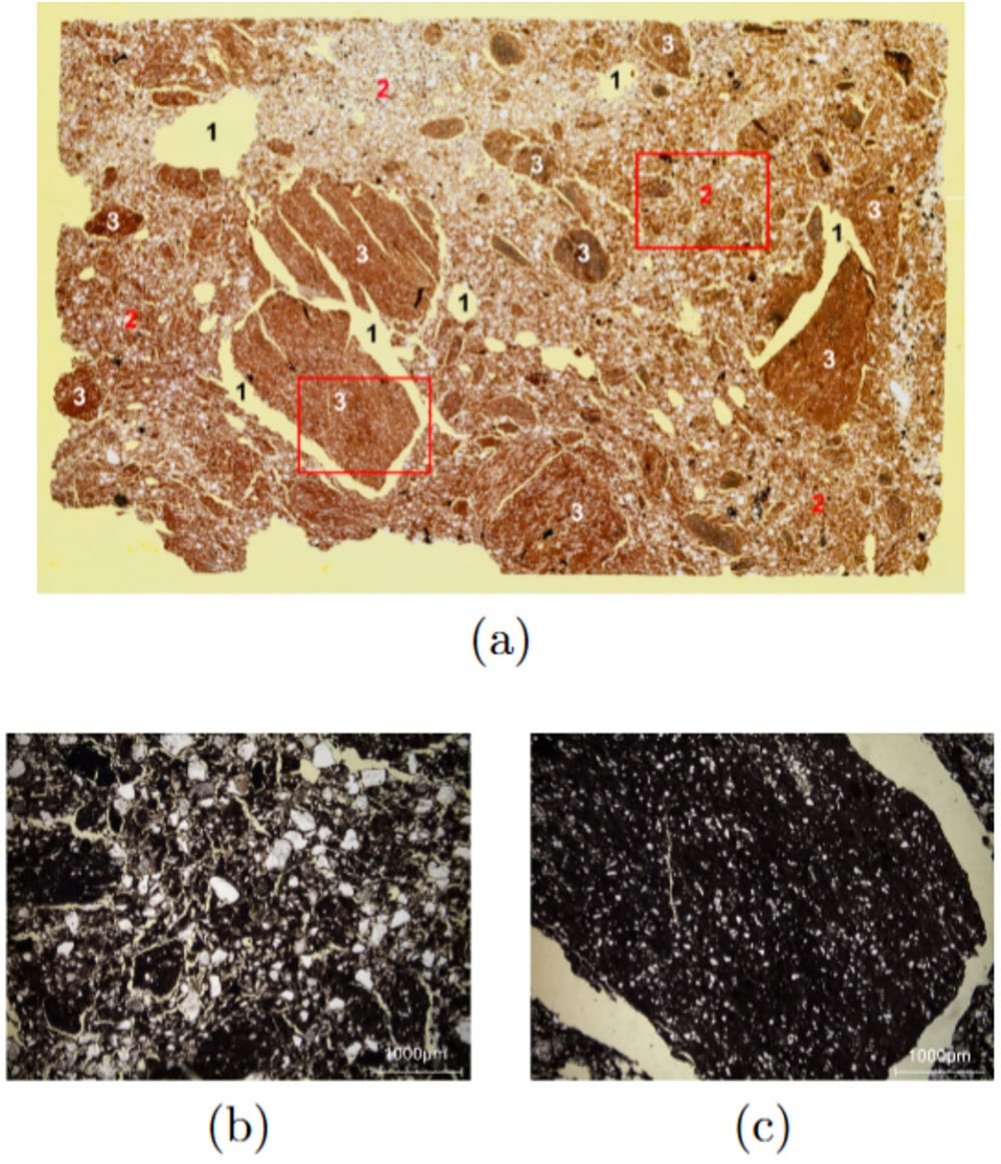
(**a**) General view of the thin section of the brick (single shot taken using a light table and a camera). Large rounded voids and shrinkage cracks are labelled as ‘1’, the clay matrix is labelled as ‘2’, and the big clay-rich inclusions are labelled as ‘3’. The red frames indicate the locations of the detailed views shown in (**b**) and (**c**). (**b**) Detail view on the clay matrix in the thin section (single polarized light). Minute shrinkage cracks in the clay matrix can be seen in yellow, while quartz grains in white are nonporous. (**c**) Detail view on a big clay-rich inclusion (single polarized light) surrounded by a large shrinkage crack (in yellow).

The Brick shows a heterogeneous pore system (see Fig. 11(a) for a general view of the related thin section). The largest pores corresponds to millimeter-sized voids and shrinkage cracks wider than 0.1 mm (indicated as ‘1’ in the Fig. 11(a)). The fired clay matrix which include sand grains shows inherent fine porosity (indicated as ‘2’ in the Fig. 11(a)). The finest pores can be found in the unmixed clay lumps which lack sand grains (indicated as ‘3’ in Fig. 11(c)). This microstructural heterogeneity aligns well with the moisture maps (see Fig. 4). Throughout increasing saturation, the highest water content is observed in the porous zones like tapered voids, cracks and the fired clay matrix. The unmixed clay lumps consistently shows low water saturated due to the extremely fine pores which are not voluminous enough to contain large amounts of water.

**Fig. 12:**
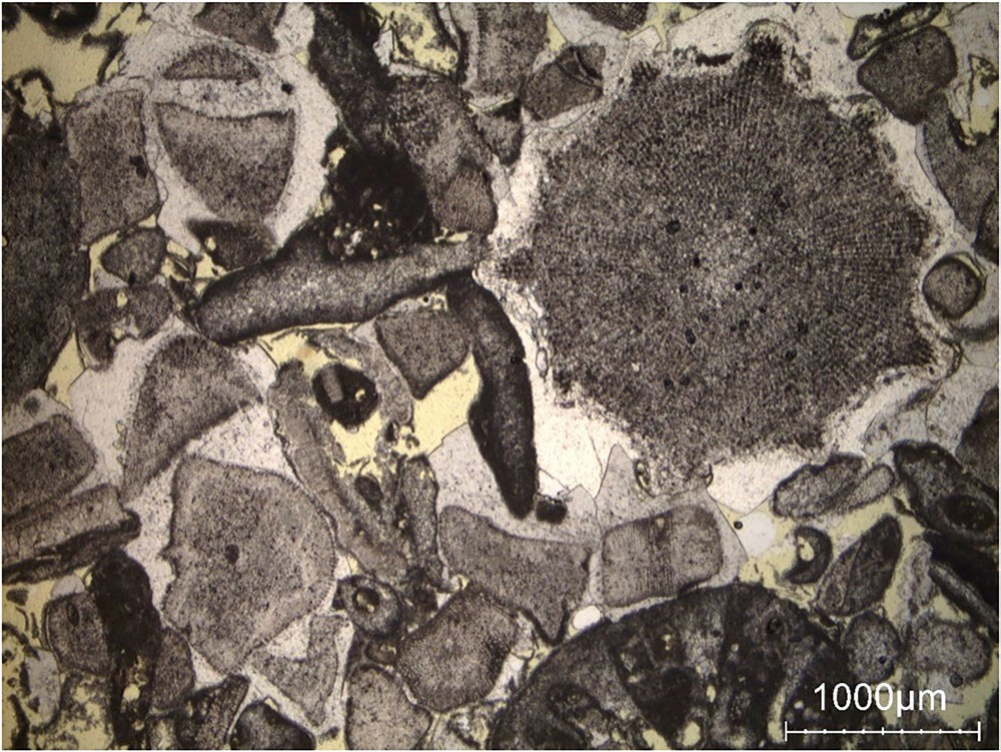
Detail view showing the general appearance of Euville stone under a polarizing microscope (single polarized light). The intergranular pores can be seen in yellow. In this picture, most of the stone grains appear in grey and are covered by overgrowths of calcite cement (in white). Rest of the stone grains appear in black.

Euville limestone, (see Fig. 12 for a detailed view) is primarily composed of crinoidal grains wih syntaxial calcite cement. The porosity is mainly intergranular (highlighted in yellow) and relatively unimodal. This corresponds closely with the moisture maps (see Fig. 5, showing progressive water saturation of the intergranular pores, followed by gradual saturation of the intragranular pore network in tandem with the intragranular pores of the crinoidal grains and their syntaxial cement.

**Fig. 13:**
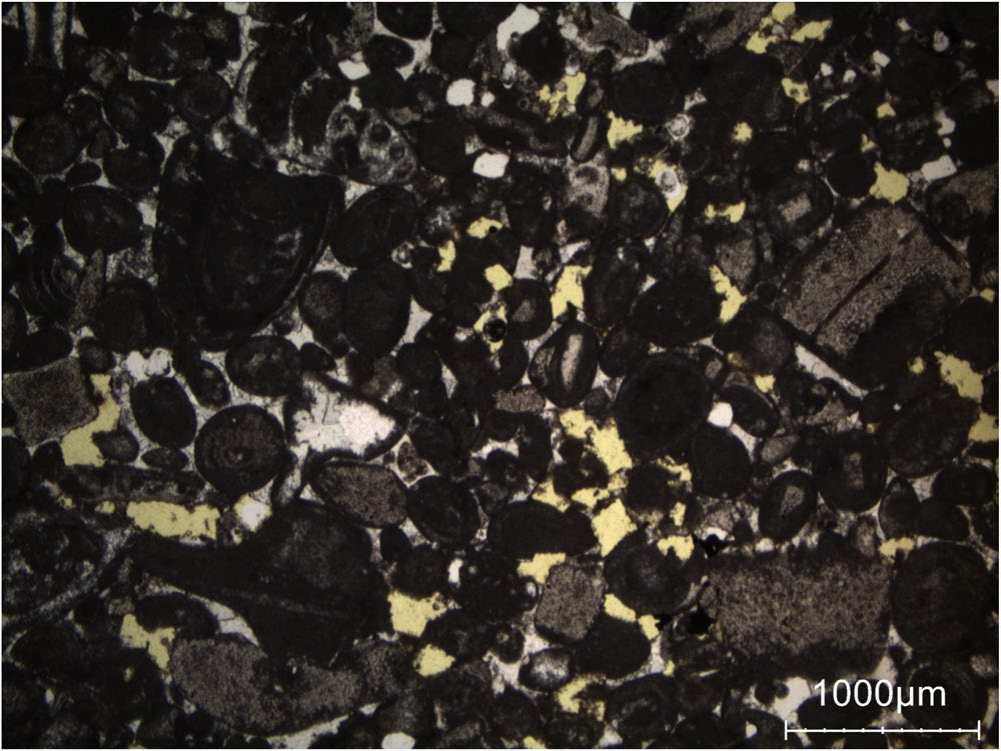
Detail view showing the general appearance of Massangis stone in the thin section (single polarized light). The more compact zones correspond to places where the stone grains (mainly oolites, in dark grey or black) are almost entirely cemented by the calcite binder (in white). The less compact zones show large macropores (in yellow) and a lower content of intergranular calcite binder.

In Massangis limestone, more porous zones alternatve with more dense zones (see Fig. 13), which have previously been described^39^. In dense zones, the natural cement fills most of the intergranular pore space, resulting in a relative low and fine inter- and intragranular porosity. In more porous zones, dedolomitization has led to larger moldic and intergranular macropores, recognizable by their rhomboedric shape (in yellow on the figure). This diagenetic zonation results in zones with different moisture content, which is clearly observable in the moisture maps (see Fig. 6), with larger amounts of water concentrated in the moldic, more porous zones.

**Fig. 14:**
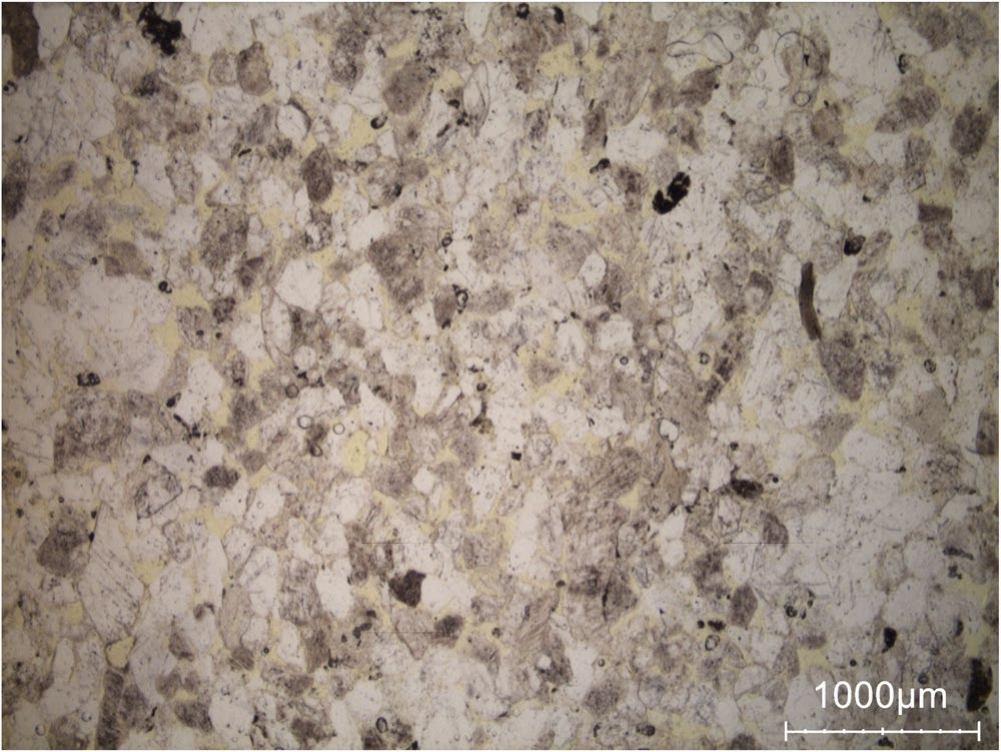
Detail view showing the general appearance of Neubrunn sandstone in the thin section (single polarized light). The intergranular pores are visible in yellow on the picture. Quartz grains (nonporous) appear white. Feldspar grains and rock fragments (microporous) are light grey.

In Neubrunn sandstone (see Fig. 14) for a detailed view), the main porosity type corresponds to intergranular pores (highlighted in yellow), susceptible to grain size variations according to its bedding. This corresponds to the moisture maps; with progressive water saturation in the intergranular pores, however, with rock lamina becoming pronounced as lamina with finer grains and thus pores are expected to retain most water at low overall mosisture content. However, with increasing overall moisture content, lamina with larger pores progressively become filled with a proportional larger volume of water (see Fig. 7

**Fig. 15:**
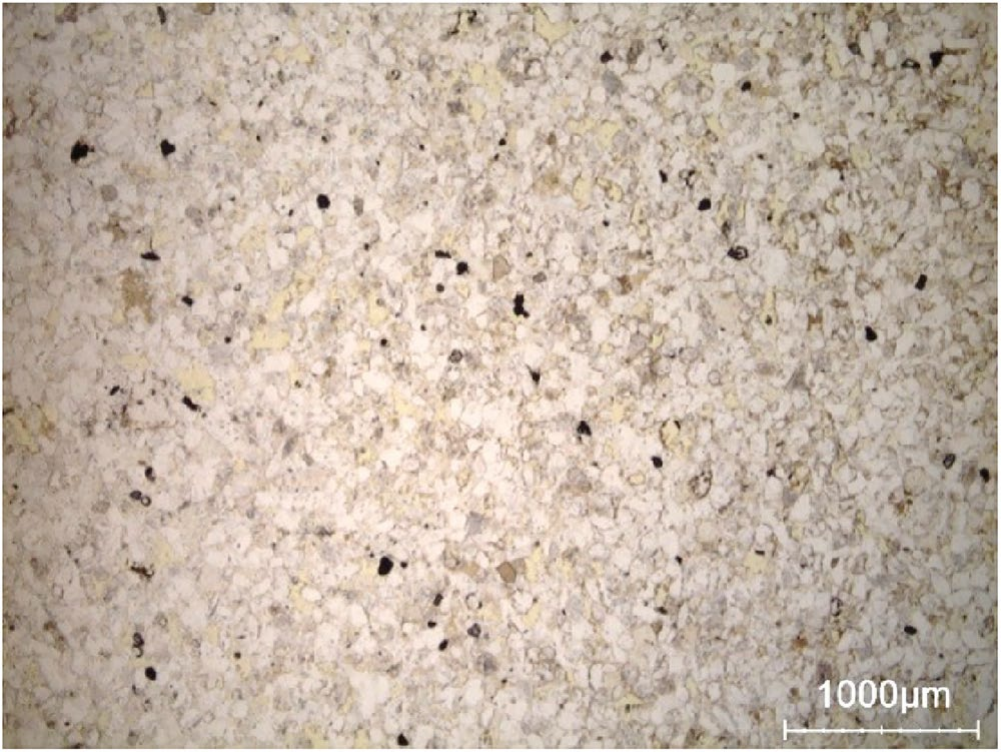
Photomicrograph showing the general appearance of Obernkirchen sandstone under a polarizing microscope (single polarized light). The intergranular pores are visible in yellow on the picture. Quartz grains (nonporous) appear white. Rock fragments (microporous) appear in light grey.

Obernkirchen sandstone (see Fig. 15) for a detailed view), is not very distinct from Neurbrunn sandstone, showing an intergranular porosity (highlighted in yellow) but less distinct layering. Petrographic characterization of Obernkirchen sandstone shows that water is primarily accommodated in the intergranular pore network, accounting for the largely homogeneous moisture distribution at all saturation levels (see Fig. 8). The limited presence of intragranular pores within rock fragments explains the minimal additional moisture uptake, supporting the consistency between the mapped moisture patterns and the stone’s internal pore structure.

**Fig. 16:**
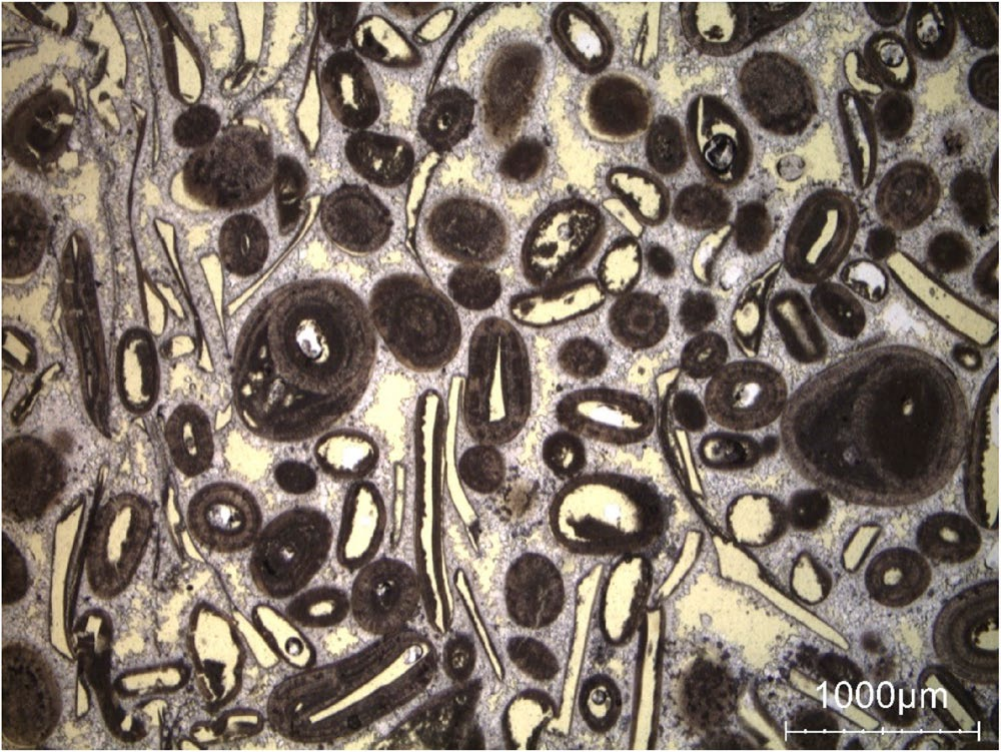
Detail view showing the general appearance of Savonnières stone under a polarizing microscope (single polarized light). The large pores (intergranular and intragranular) appear in yellow. Finer pores are present in the calcite cement (intercrystalline, in white) and inside the oolites (intragranular, in dark grey).

Savonnières limestone has a multimodal pore size distribution (see Fig. 16) for a detailed view with explanation). From large to small size, it considers well connected intergranular and poorly connected intragranular pores, complemented by smaller intergranular pores between the dogtooth cement and the finest intragranular pores in the micritic structure of the ooid grains. In addition, the stone shows layering on the macroscale. This causes the initial uneven water distribution and the eventual homogenization at higher saturation levels (see Fig. 9).

In summary, hyperspectral imaging-derived moisture maps are rigorously validated through complementary quantitative and qualitative approaches. Quantitative comparison with gravimetric measurements shows strong agreement, with low RMSE values (1-2 g/g  × 100), confirming reliable mean moisture estimates across heterogeneous samples. As observed in the moisture maps, Brick exhibits sequential wetting, with moisture first occupying large shrinkage cracks before spreading into the clay matrix and fine inclusions, consistent with its petrographic pore structure. In Massangis limestone, the patchy zones of higher moisture content correspond to large, poorly cemented areas revealed in thin sections, while Euville limestone shows progressive wetting from intergranular pores to intragranular and intercrystalline spaces, reflecting the mapped saturation patterns. Neubrunn and Obernkirchen sandstones demonstrate moisture preferentially following intergranular pores and laminae, and in Savonnières limestone, heterogeneous absorption aligns with large pores, as indicated by the maps. Together, the quantitative and petrographic validations confirm that the moisture maps faithfully capture pore-scale water distribution and material-specific moisture dynamics, demonstrating the coherence and reliability of the dataset. In future work, additional stone and brick types will be studied, hyperspectral microscopy will be applied to cylindrical sub-samples, and pore-scale moisture behavior will be benchmarked against MIP.

### Consent for publication

The authors confirm that they consent to the publication of this dataset in *Scientific Data*. Users of the data are required to provide appropriate credit and citation to this publication.

## Data Availability

The full dataset is openly available through the Zenodo repository and can be accessed via 10.5281/zenodo.17726161

## Code availability

The code used to estimate the moisture content of the moist samples and generate the corresponding moisture maps is openly accessible on GitHub: https://github.com/Danish-ac98/Hyperspectral_Dataset.
